# Estimates that cannot fail visibly: a current debate on measuring child mortality as household surveys contract

**DOI:** 10.1080/16549716.2026.2752841

**Published:** 2026-10-26

**Authors:** Augustus Osborne

**Affiliations:** Health Systems, Institute for Development, Freetown, Sierra Leone

**Keywords:** Demographic and Health Surveys, civil registration and vital statistics, data availability, Bayesian modelling, sub-Saharan Africa

## Abstract

Global health has an argument on its hands that it has not yet had openly. For most of the countries that carry the burden of child deaths, the published under-five mortality rate is not a measurement. It is a model output built largely from birth histories collected in household surveys, because only about a tenth of deaths are registered in the World Health Organization African region. That input stream contracted sharply in 2025, when United States funding ended for the survey programme that had supplied about half of the child mortality data points for sub-Saharan Africa; interim funding now sustains only selected services. Three positions can be discerned in response, two of them largely implicit: that the estimation model will absorb the loss, that the contraction is a reason to accelerate civil registration, and that the survey stream must be restored. I argue that none can be settled on present information, because the evidence behind each estimate is published apart from the estimate and from the products that use it, so the figures look the same whether or not new evidence has arrived. I propose three steps in order of priority: print the age, number and type of the data sources beside every country estimate; direct scarce survey funding first to countries whose evidence is oldest and whose burden is largest; and make sample-based mortality registration a nationally owned and financed function of the state, as the bridge to complete civil registration.

## Paper Context

- **Main findings:** Published child mortality estimates for countries without complete death registration look the same whether or not new survey evidence has arrived.
- **Added knowledge:** The age and number of data sources behind each national estimate, already held by the estimating agencies, would show whether the model is absorbing or concealing the loss of survey data.
- **Global health impact for policy and action:** Estimates should carry the age and type of their data, survey funding should go where evidence is oldest, and governments should own sample-based mortality registration until civil registration is complete.

## Background

In 2024, an estimated 4.9 million children died before their fifth birthday, 2.3 million of them in the neonatal period [1]. These figures rank countries and direct financing. For most countries that carry the burden, they are not measurements.

For much of sub-Saharan Africa and southern Asia, child mortality is estimated from household survey birth histories fed into the model of the United Nations Inter-agency Group for Child Mortality Estimation (UN IGME), a Bayesian spline with bias adjustment [2]. About 40% of the world’s deaths go unregistered, and in the World Health Organization African region only about 10% of deaths are registered [3]. There, the survey does the work of the register (Figure 1).

**Figure 1.**
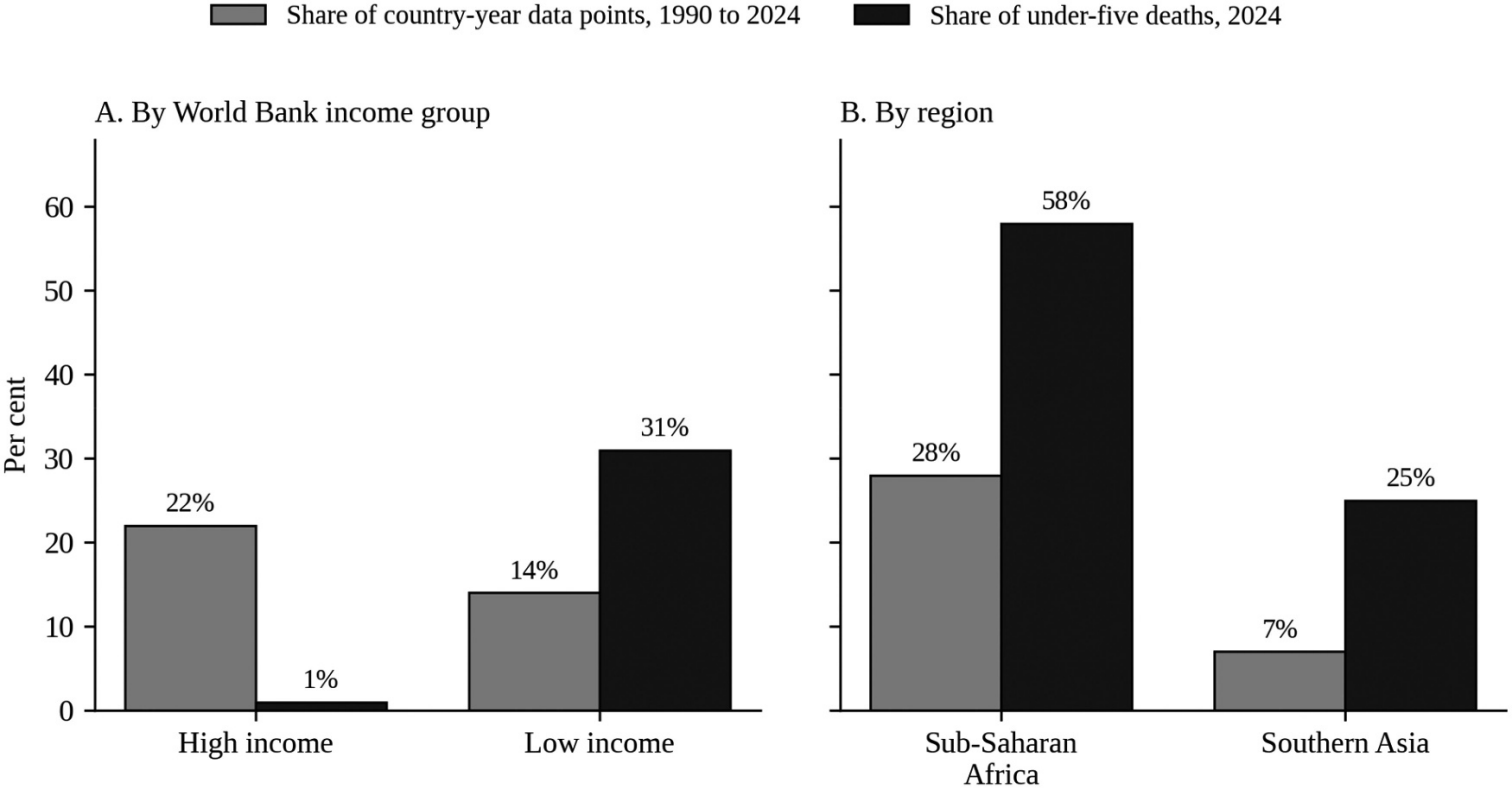
Share of country-year data points in the UN IGME database, 1990 to 2024, and of global under-five deaths in 2024, by World Bank income group (A) and region (B), as published by UN IGME [1].

The Demographic and Health Surveys (DHS) supplied 16% of the child mortality data points used for the United Nations World Population Prospects, and 50% in sub-Saharan Africa, and were the only source in at least one year for 85 countries [4]. United States funding ended in 2025, with surveys under way or ready to start in 23 countries [5]. Interim philanthropic funding now covers selected services only [6,7]. Meanwhile official development assistance fell 23.1% in real terms in 2025 [8], and development assistance for health fell from 80.3 billion United States dollars in 2021 to an expected 38.4 billion in 2025, in 2023 dollars [9].

### The state of the argument

The response has settled into three positions. Only the third has been argued at length in print; the first two are my reconstruction of stances implicit in practice and informal discussion, stated as strongly as I can.

The first is that not much has happened. The model borrows strength across time and countries and widens its intervals where data are thin [2], and estimates for many countries already ‘rely on extrapolation based on trends from prior years’ [10]. On this view, the method absorbs a lost survey round; that is what it is for.

The second is that surveys were never the right instrument. Civil registration underpins every country with reliable child mortality statistics and has had a decade of international attention [11]. Proposals to fill the gap from registration and routine health information systems are aired more often in meetings than in print [12], and a milder version holds that strengthening these systems would reduce dependence on surveys [5].

The third is that the survey stream must be restored quickly, because nothing else can produce a national under-five mortality rate for most high-burden countries before the next decade of child survival financing is allocated [5,13].

My position cuts across all three. The dispute proceeds without an account, beside each country’s estimate, of how much evidence stands behind it and how old it is. Without it, the first position cannot be tested, since nobody can see whether the model is absorbing the loss or concealing it; the second cannot be timed; and the third cannot be targeted.

## Why the estimates cannot signal their own failure

A model that borrows strength across time and countries produces a number for every country every year. The design is sound, but the headline figure for a country with no survey since 2019 looks like the figure for a country surveyed last year. The evidence is not hidden. UN IGME publishes its input data [2] and summarises their recency: in the 2025 round, 38 countries had empirical under-five data for 2024, 162 required extrapolation, and the global mean extrapolation period was 4.5 years [14]. What is missing is the information’s place. It sits in methods papers, summaries and a data portal’s country charts, and does not travel with each figure into the rankings, dashboards and progress trackers that decision makers use, a risk others have raised [15,16].

Thin data has produced divergent answers before. In 2011, UN IGME and the Institute for Health Metrics and Evaluation (IHME) estimates for 2010 differed by more than 10 deaths per 1,000 live births in a fifth of countries, mainly because the groups used different data [17]. Collaboration may since have narrowed the gap, but agreement between models fed largely the same data is not evidence that either is right; I know of no later decomposition.

The intervals widen with time since the last observation. Validation is partly reassuring. With data collected after 2005 withheld, about 92% of left-out observations fell within 90% prediction intervals, though predictions ran about 11% high at the median [18]. In the neonatal model, with each country’s latest survey withheld, 92% of left-out observations fell within 90% intervals, but only 77% of post-2005 estimates from reduced data lay within the full-data 80% bounds [19]. These tests covered years of steady decline; none tested a decade-long gap opening while falling aid could bend the trends being extrapolated.

The damage will also arrive slowly. A large, full birth history supports single-year estimates for about a decade before the survey [20], so a cancelled round removes the newest evidence first. Its effect will grow over several rounds, unseen, the worst profile for anyone deciding when to act.

The test that separates the first position from mine can be run now. UN IGME rounds are archived, and revisions after new data are documented: between the 2012 and 2013 rounds, estimates for 2011 changed by more than 10 deaths per 1,000 in 33 countries, in ten because of new data [21], and new surveys in ten countries differed from UN IGME and IHME child mortality predictions for 2015 by a median of 15 and 19% [15]. Comparing each country’s estimate before and after a delayed survey, against the length of the gap, would show how far extrapolations drift and whether the intervals anticipated it. To my knowledge no analysis by length of gap has been done.

### The first position: the intervals already carry the warning

The strongest defence of current practice is that nothing is concealed: the intervals widen as data thins, for any careful reader to see. I do not dispute the statistics.

In my experience, rankings, dashboards and country briefings carry the point estimate and drop the interval. Nor does an interval show the age of the last observation, or the quantity a minister needs; by the time it is wide enough to notice, the years for investment have passed.

### The second position: registration rather than surveys

The case for civil registration is the strongest of the three. I accept its destination and dispute its timetable. Registration is the only continuous, universal, state-owned record, and countries have made real progress towards it [11].

Roughly a fifth of the world’s population lives in the 43 countries whose civil registration systems perform least well [22]. No plausible rate of improvement closes the gap within the coming decade of child survival financing. Treating the two as alternatives produced the present position, in which the interim instrument was defunded before the permanent one existed. They are a sequence, and a sequence must be financed as one thing.

### Other sources

UNICEF’s Multiple Indicator Cluster Surveys, untouched by the 2025 termination, are a primary source of birth histories [2] and can partly fill the gap, but they run on their own schedules and face threatened cuts to UNICEF’s funding [5]. Censuses supply summary birth histories for indirect estimation [23], about once a decade. Health and demographic surveillance sites and the Child Health and Mortality Prevention Surveillance network record deaths continuously, but in catchment areas that are not nationally representative [24–26]. Some donors and host governments are completing selected surveys [6]. None of these yet produces a national rate every few years for most countries that lost their survey.

## Where this leaves the argument

Resources are scarce, so order matters: the first step is cheap and enables the others, the second targets the remaining survey money, and the third changes who owns the measurement.

Every country figure should carry, at the same prominence as the rate, the year of the most recent data source (survey, census or registration year, not reference year), the number of independent sources in the past decade, whether each is nationally representative, and the years since the last reference period with direct data. Raw counts of data points would mislead, since one survey yields many. A model-based measure, such as the ratio of posterior to prior interval width, would be better still. The agencies hold this information, so the change is cheap, though not free for reports and downstream products.

With the evidence base printed, financiers can rank countries by the age of their latest source and the child deaths at stake, and spend first where both are high. The programme cost about 47 million United States dollars a year in nominal terms [5], small against the flows it informs, and full domestic substitution is not realistic soon: 27 of 47 African countries are highly or very highly vulnerable to cuts in external health financing [27].

The change I would pursue now, as a researcher in a country that lives with these estimates, is to stop treating national mortality measurement as a project that arrives with a donor and leaves with it. Sample registration does not need a decade. Mozambique’s system covered 828,663 people using verbal autopsy; its under-five rate, 80 per 1,000, lay close to the UN IGME estimate made without it, 74 (interval 48 to 107) [26]. Sierra Leone’s sample registration system completed a nationally representative first round covering about 343,000 people [28]. Both were largely donor-financed, which is the fragility to remove. Verbal autopsy also assigns causes with uncertainty, needs local calibration and depends on maintained sampling frames [26,29]. The decisive change is institutional: each high-burden government places sample registration in its statistics office on a domestic budget line, partners co-finance on a declining schedule as the domestic share grows, and the national series becomes the reference that global models must reconcile with and explain. That moves the centre of measurement to the country, within the decade that complete registration cannot.

## Conclusion

The concern is not that the estimates are wrong but that no one can tell whether they are, and none of this settles which instrument deserves the next dollar. A measurement system that cannot fail visibly cannot tell anyone when to invest in it.

## Data Availability

Not applicable. No new data were created or analysed.
